# Quantitative Comparison of Age‐Related Development of Oral Functions During Growing Age

**DOI:** 10.1002/cre2.70033

**Published:** 2024-11-12

**Authors:** Kiichiro Mizokami, Syunnosuke Tohyama, Hiroyuki Kanzaki, Yoji Sasaki, Mao Katayama, Minami Seki, Haruna Rikitake, Syoutaro Ueda, Toshiko Sekiya, Hiroshi Tomonari

**Affiliations:** ^1^ Department of Orthodontics, School of Dental Medicine Tsurumi University Kanagawa Japan

**Keywords:** aging, bite force, development, lip pressure, masticatory efficiency, maturation, occlusal contact area, oral function, tongue pressure

## Abstract

**Objectives:**

This cross‐sectional study aimed to investigate the age‐related development of five major oral functions—tongue pressure, lip pressure, masticatory efficiency, bite force, and occlusal contact area—in subjects aged 5–20 years.

**Material and Methods:**

Ninety‐two subjects were divided into four age groups: 5–8, 9–12, 13–16, and 17–20 years. Oral functions were evaluated using standardized methods, including Chew Check Gum for assessing masticatory efficiency, Dental Prescale II for measuring occlusal force and contact area, JMS tongue pressure measuring device for evaluating tongue pressure, and Ripple‐Kun for measuring lip pressure.

**Results:**

Occlusal contact area, maximum bite force, tongue pressure, and masticatory efficiency gradually increased with age, whereas lip pressure remained stable. The occlusal contact area temporarily decreased between 5 and 12 years of age, likely due to the replacement of erupting lateral teeth. Some differences were observed between males and females; however, overall trends in oral function parameters were similar. Spearman's rank correlation analyses revealed significant positive correlations between age and occlusal contact area, bite force, tongue pressure, and masticatory efficiency.

**Conclusions:**

Oral function parameters exhibited different age‐related development patterns. Occlusal contact area, maximum bite force, tongue pressure, and masticatory efficiency gradually increased with age. Interestingly, the occlusal contact area tended to reduce temporarily between 5 and 12 years of age due to the replacement of erupting lateral teeth. Conversely, lip pressure was almost stable during this period. Our findings provide valuable insights into the developmental patterns of oral functions during the growing years.

## 1 Introduction

The mouth is an important organ responsible for essential functions, such as feeding, chewing, swallowing, articulation, taste, speech, and saliva secretion; these oral functions are performed through coordination among the tongue, lips, and masticatory muscles (Fujii 1971). The development of proper oral function is essential for a child's healthy growth. Inadequate oral function development can lead to poor nutritional intake because of chewing disorders, which in turn can negatively affect overall development (Sato and Yoshiike, 2011). Previous studies have shown an association between oral function and obesity (Araujo et al. 2016; Ohno et al. 2020; Sun et al. 2016). Oral dysfunctions can also interfere with communication skills, causing poor pronunciation and dysarthria (Yamaguchi et al. 1994). Furthermore, improper oral function can lead to abnormal maxillofacial growth (Yamaguchi and Sueishi, 2003). Proper oral function development during growth also contributes to the establishment of good eating habits in the future (Fujita 2022; Monda et al. 2021; Sogabe et al. 2010). A high mastication ability allows the consumption of a variety of foods and contributes to a well‐balanced diet (Le Révérend, Edelson, and Loret, 2014). Proper oral function also protects against caries and gum diseases (Rapeepattana, Suntornlohanakul, and Thearmontree, 2019). Several reports have indicated that the development of these functions starts in infancy and reaches levels nearly equivalent to those of adults by adolescence (Almotairy et al. 2018; Almotairy, Kumar, Noirrit‐Esclassan, et al. 2020; Almotairy, Kumar, Welander, et al. 2020; Fujita et al. 2018; Ichikawa et al. 2016; Kamegai 2005; Owais, Shaweesh, and Abu Alhaija, 2013; Usui et al. 2007).

The development of malocclusion is a gradual process that often begins in early childhood, as evidenced by longitudinal studies (Luzzi et al. 2017). This progressive nature underscores the importance of understanding the interplay between malocclusion and oral function in pediatric populations. Recent research has illuminated several critical aspects of this relationship. First, malocclusion in children has been shown to significantly impact masticatory efficiency and maximum bite force (Alshammari et al. 2022; Toro et al. 2006). These findings suggest that occlusal discrepancies may have functional consequences even at young ages, potentially affecting nutritional intake and overall oral health. Furthermore, a study on children aged 5–12 years revealed that mouth‐breathing, which is often associated with malocclusion, correlates with reduced tongue pressure (Azevedo et al. 2018), indicating that breathing patterns and tongue function may influence occlusal development from an early stage. Of particular interest is the relationship between open bite malocclusion and perioral muscle function. Children with open bite have been observed to exhibit weaker lip closure forces and difficulty in achieving proper lip seal (Ueda et al. 2002; Yata et al. 2001). These observations are crucial, as adequate lip competence is essential for various oral functions, including speech articulation and the maintenance of a physiological oral environment. Moreover, recent investigations have expanded our understanding of the multifaceted nature of malocclusion's impact. For instance, Choi et al. (2015) found that malocclusion severity was associated with reduced oral health‐related quality of life in children, highlighting the psychosocial implications of occlusal discrepancies. These findings collectively emphasize that the age‐related development of oral function in children is intricately linked to occlusal status. This relationship is bidirectional; malocclusion can influence functional development, and aberrant functional patterns may contribute to the progression of occlusal abnormalities. Therefore, early assessment and intervention in cases of developing malocclusion are crucial not only for aesthetic concerns but also for ensuring optimal functional development and overall oral health in pediatric populations.

Current reports on the development of oral function with age are as follows. Masticatory efficiency has been reported to improve between 6 and 17 years of age, suggesting that this change is influenced by the loss of deciduous teeth in the late mixed dentition period (Barrera et al. 2011). Furthermore, the evaluation of three groups per Hellman's dental age (IIIA, IIIB, and VA) suggested that there was no significant change in masticatory ability during the mixed dentition stage and that masticatory ability was promoted after the IIIB stage (Fujita et al. 2018). Maximum bite force has been reported to increase between 7 and 17 years of age, with maximum bite force in the incisors peaking at 14.3 years for females and 15.3 years for males and maximum bite force in the molars peaking at 16 years for both sexes (Roldán et al. 2016). Maximum tongue pressure increases with increasing age in boys, whereas it decreases in teenage girls (Guo et al. 2021). Lip pressure was investigated in children aged 3–12 years, and two phases were observed: a developmental (3–6 years) phase and a stable (7–12 years) phase (Saitoh et al. 2017). However, the detailed developmental process of each oral function according to age has not been fully elucidated.

This study focused on five major oral functions—tongue pressure, lip pressure, masticatory efficiency, bite force, and occlusal contact area—and examined their development during the growing stage. By clarifying the developmental pattern of each function, we aim to provide basic data to promote the healthy growth of children. Moreover, the findings of this study can aid in planning measures to appropriately support oral function development.

## 2 Materials and Methods

### 2.1 Ethical Issues

The protocol for this study was approved by the Ethics Committee of Tsurumi University, School of Dental Medicine (approval numbers: 1812 and 1833). This retrospective clinical study adhered to the ethical guidelines for medical and health research involving human subjects in Japan. We implemented an opt‐out consent process, in compliance with these guidelines. Information regarding the study was prominently displayed on the hospital's bulletin boards, providing patients with the opportunity to decline participation and exclude their data from research use. All patients who did not exercise this opt‐out option were subsequently included in the study cohort. This approach ensured respect for patient autonomy while facilitating the conduct of valuable retrospective research.

### 2.2 Sample Size Calculation

The sample size was calculated using G Power 3.1.9.4 (Universität Kiel, Germany), with group number = 4, alpha = 0.05, power = 0.8, and effect size = 0.4 (Alam and Alfawzan 2020). The minimum sample size was computed as 76, and we included 92 subjects.

### 2.3 Subjects

Patients who visited our hospital for orthodontic treatment in October 2019 and June 2023 for the first time were included in this study. In total, 92 patients (43 males and 49 females; age: 5–20 years) were selected using the following inclusion and exclusion criteria.

#### 2.3.1 Inclusion Criteria

Inclusion criteria included the following: (1) overjet between 0 and +6 mm, (2) overbite over 0 mm and mandibular incisors not contacting palatal gingiva, (3) arch length discrepancy less than −4 mm, (4) skeletal class I and average mandibular plane angle judged by Japanese standard value (Japanese Society of Pediatric 1995), and (5) lateral Me deviation of 2 mm or less.

#### 2.3.2 Exclusion Criteria

Exclusion criteria included the following: (1) those with crossbite, (2) those with congenital diseases, (3) those with temporomandibular joint disorders (TMDs), and (4) those with permanent missing teeth.

All participants were examined for orthodontic treatment, and the test results of oral function tests were used for this study.

### 2.4 Group Setting

Subjects were divided into four groups according to age: Group 1 (age: 5–8 years; 22 subjects, 13 females and 9 males); Group 2 (age: 9–12 years; 22 subjects, 10 females and 12 males); Group 3 (age: 13–16 years; 30 subjects, 16 females and 14 males); and Group 4 (age: 17–20 years; 18 subjects, 10 females and 8 males). The demographic information of each group is presented in Table 1.

**Table 1:** The demographic information of each group.

Group	Age				Number of participant			Dental age					
	Range	Mean ± SD	Male	Female	All	Male	Female	ⅡC	ⅢA	ⅢB	ⅢC	ⅣA	ⅣC
1	5–8	7.6 ± 0.6	7.6 ± 0.5	7.6 ± 0.6	22	9	13	2	15	5	0	0	0
2	9–12	10.3 ± 1.1	10.5 ± 1.2	10 ± 0.9	22	12	10	0	6	14	2	0	0
3	13–16	14.6 ± 1.2	14.9 ± 1.3	14.4 ± 1.1	30	14	16	0	0	0	11	19	0
4	17–20	18.9 ± 1.0	18.9 ± 0.8	19 ± 1.2	18	8	10	0	0	0	0	17	1

*Note:* There is no difference in the male‐to‐female ratio among groups.

### 2.5 Evaluation of Oral Functions

Oral function evaluation methods have been described elsewhere (Sasaki et al. 2023). Masticatory efficiency was evaluated using color‐changeable chewing gum (Chew Check Gum, Oral Care Inc., Tokyo, Japan) (Komagamine et al. 2011). After rinsing for 15 s, subjects chewed the gum for 60 cycles and then flattened it into a circular shape. The color change of the gum was used as an indicator of masticatory efficiency and measured using a colorimeter (CR‐20, Konica Minolta Inc., Tokyo, Japan). Measurements were performed three times each in the order of free chewing, right‐side chewing, and left‐side chewing, with 3‐min rest periods between each chewing trial. The mean value of free chewing was used for analysis.

Occlusal force and contact area were assessed using Dental Prescale II (GC Corporation, Tokyo, Japan) (Horibe et al. 2022). Subjects practiced biting on a training film inserted intraorally for 3 s with their molars. Subsequently, the actual measurement was conducted using the Prescale film. A specialized scanner was used to analyze the film and calculate the maximum occlusal force and contact area.

Tongue pressure was measured using the JMS tongue pressure measuring device (JMS Co. Ltd., Hiroshima, Japan) (Utanohara et al. 2008). Subjects were instructed to press their tongue firmly against the palate for 7 s with the probe placed on the tongue. After one practice attempt, three measurements were taken, and the average was recorded as tongue pressure.

Lip pressure was evaluated using the Ripple‐Kun device (Shofu Inc., Tokyo, Japan) (Ueda et al. 2002). Subjects were instructed to insert the button into the oral vestibule and close their lips. The button was then pulled horizontally, and the force at which it exited the oral vestibule was measured. Following one practice trial, three measurements were taken, and the average was calculated as lip pressure.

These tests were calibrated before the use and performed by trained investigators, and the reproducibility of each test was high. The intraclass correlation coefficients (ICC) values were as follows: 0.73 for masticatory efficiency, 0.95 for occlusal force, 0.86 for occlusal contact area, 0.83 for lip pressure, and 0.96 for tongue pressure (Sasaki et al. 2023).

### 2.6 Statistical Analysis

The normality of the obtained data was analyzed using the Kolmogorov–Smirnov test. The chi‐square test was used to compare the male‐to‐female ratio in each group. Kruskal–Wallis test followed by pairwise tests with Bonferroni adjustment and the Steel–Dwass test were used to compare oral function parameters among groups. Spearman's rank correlation coefficient was used to examine the correlation between age and the different oral functions. Each oral function parameter within groups was compared between males and females using the Mann–Whitney *U* test. All statistical analyses were performed using SPSS Statistics version 27.0 (IBM, Tokyo, Japan) and web‐based Steel–Dwass tests (http://www.gen-info.osaka-u.ac.jp/testdocs/tomocom/s-d.html). A *p* value of < 0.05 was considered statistically significant.

## 3 Results

### 3.1 Demographic Information of the Subjects

The numbers of subjects in Groups 1, 2, 3, and 4 were 22, 22, 30, and 18, respectively (Table 1). There were no significant differences in the male‐to‐female ratio among these groups. Regarding the dental age in each group, Groups 1 and 2 were dominated by IIIA and IIIB, respectively. The percentage of people with dental age IIIB in Group 2 appeared to be different between males (50%) and females (80%), although the difference was not statistically significant (*p* = 0.204). Group 3 consisted of IIIC and IVA, and Group 4 was dominated by IVA.

### 3.2 Changes in Occlusal Contact Area

**Figure 1:**
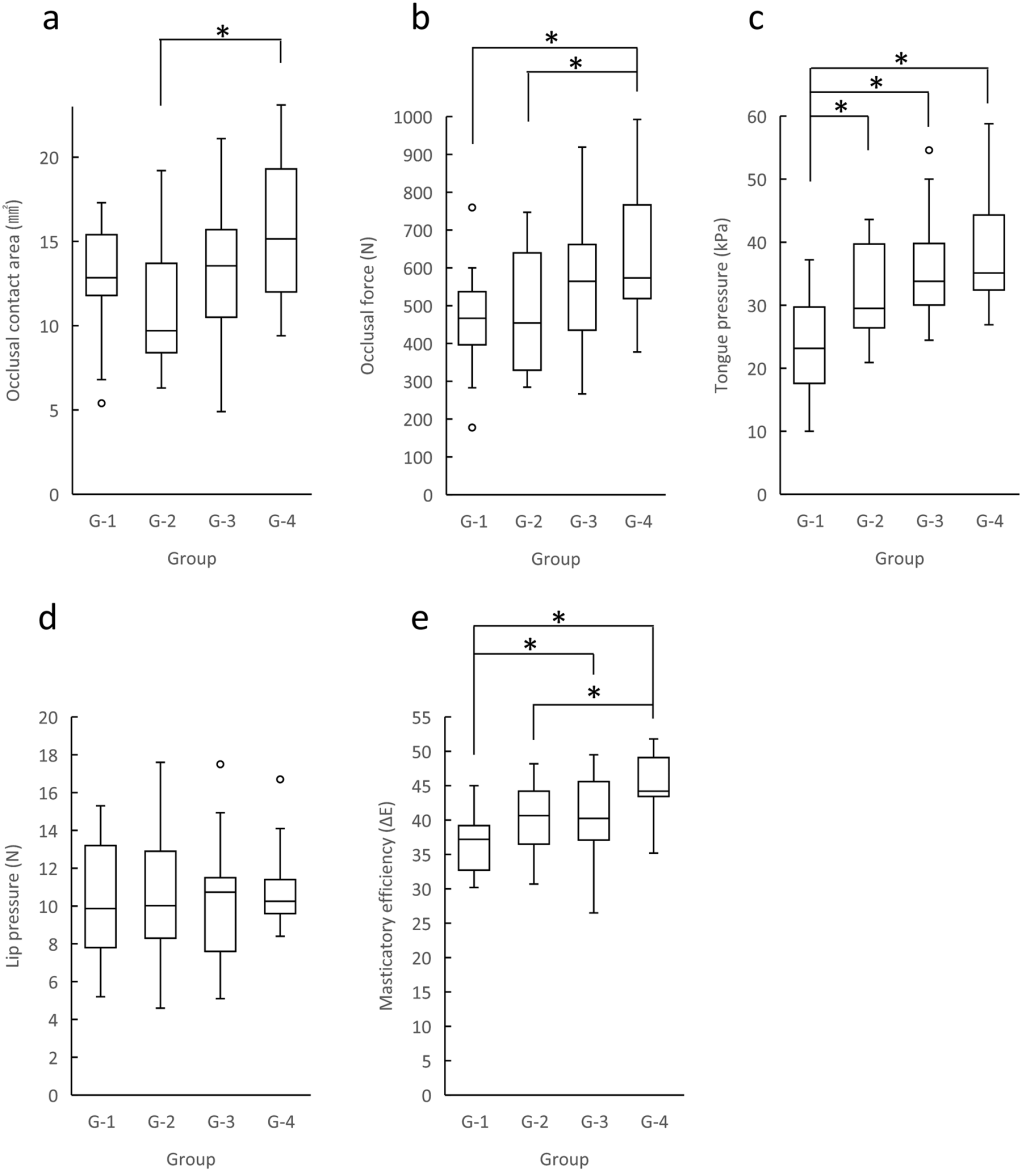
Changes in oral function parameters. The oral function parameters of each group, occlusal contact area (a), occlusal force (b), tongue pressure (c), lip pressure (d), and masticatory efficiency (e) are expressed as median, 25th percentile, 75th percentile, maximum, and minimum values. *p < 0.05.

Occlusal contact area exhibited a statistically significant difference between Groups 2 and 4 (Figure 1a). To further elucidate the relationship between age and occlusal contact area, Spearman's rank correlation analyses were performed (Table 2). Analysis using whole groups exhibited a statistically significant but slight positive correlation (*r* = 0.284). Negative correlations were observed in Groups 1 and 2; however, they were not statistically significant (*r* = −0.172, *p* = 0.263). In contrast, analyses performed in Groups 2, 3, and 4 revealed a significant positive correlation (*r* = 0.452, *p* < 0.001). These results revealed that the occlusal contact area was relatively stable in Groups 1 and 2 and increased in Groups 2, 3, and 4.

**Table 2:** Correlation analysis between each oral function and age.

		Analyzed groups				
		G1 ~ 4	G1 ~ 2	G2 ~ 3	G3 ~ 4	G2 ~ 4
Occlusal contact area	*ρ*	0.284**	‐0.172	0.392**	0.356*	0.452**
	*p* value	0.006	0.263	0.035	0.013	< 0.001
Occlusal force	*ρ*	0.410**	0.128	0.294*	0.292*	0.378**
	*p* value	< 0.001	0.408	0.016	0.044	0.001
Tongue pressure	*ρ*	0.550**	0.437**	0.333*	0.306*	0.366**
	*p* value	< 0.001	0.003	0.016	0.034	0.002
Lip pressure	*ρ*	0.147	0.058	0.176	0.293*	0.189
	*p* value	0.161	0.708	0.212	0.044	0.118
Masticatory efficiency	*ρ*	0.500**	0.379*	0.229	0.432**	0.387**
	*p* value	< 0.001	0.011	0.103	0.002	< 0.001

* *p* < 0.05

** *p* < 0.01.

### 3.3 Changes in Occlusal Force

There were statistically significant differences between Groups 1 and 4 and between Groups 2 and 4 (Figure 1b). Spearman's rank correlation analyses using whole groups exhibited statistically significant and considerable positive correlation (Table 2; *r* = 0.410). There were no statistically significant correlations in Groups 1 and 2; however, there were correlations in Groups 2, 3, and 4 (*r* = 0.378). These results suggest that occlusal force was relatively stable in Groups 1 and 2, but it increased in Groups 2, 3, and 4.

### 3.4 Changes in Tongue Pressure

Group 1 differed significantly from Groups 2, 3, and 4 (Figure 1c). Spearman's rank correlation analyses using whole groups exhibited statistically significant and moderate positive correlations (Table 2; *r* = 0.550). Analyses performed using Groups 1 and 2 revealed a moderate correlation (*r* = 0.437), whereas those performed using Groups 2 and 3 and Groups 3 and 4 resulted in mild correlations (*r* = 0.333 and 0.306, respectively). These results suggest that tongue pressure rapidly increased in Groups 1 and 2 and kept increasing in Groups 2, 3, and 4.

### 3.5 Changes in Lip Pressure

There was no statistically significant difference among the four groups (Figure 1d). There were no statistically significant correlations between age and lip pressure (Table 2), suggesting that lip pressure remained stable during the examined age.

### 3.6 Changes in Masticatory Efficiency

Group 1 differed significantly from Groups 3 and 4 (Figure 1e). In addition, there was a statistically significant difference between Groups 2 and 4. Spearman's rank correlation analyses using whole groups exhibited statistically significant and considerable positive correlation (Table 2; *r* = 0.500). There were no significant correlations in Groups 2 and 3; however, analyses performed using Groups 1 and 2 and Groups 3 and 4 revealed moderate correlations (*r* = 0.379 and 0.432, respectively). These results suggest that masticatory efficiency rapidly increased biphasically in Groups 1 and 2 and in Groups 3 and 4.

### 3.7 Comparison of Oral Function Parameters Between Males and Females in Each Group

**Figure 2:**
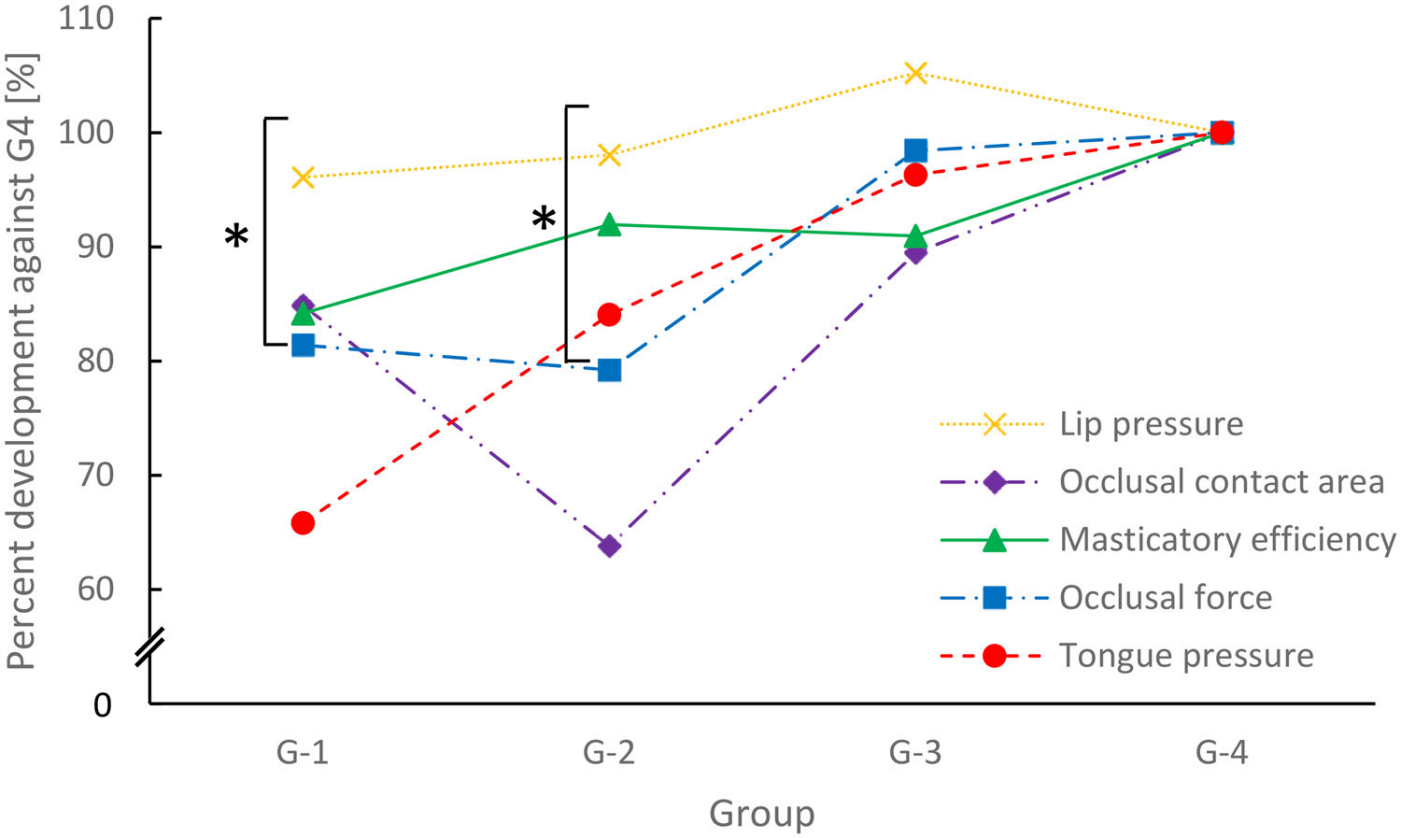
Comparison of the degree of oral function development among the four groups. The percentage development in each oral function parameter was calculated by dividing the group median value by the median value of Group 4. *p < 0.05.

We then compared each oral function parameter between males and females in each group (Table 3). There were no statistically significant differences in all parameters between males and females in Groups 1, 3, and 4. However, some parameters differed significantly between males and females in Group 2. Occlusal force and lip pressure were significantly higher in males than in females. The occlusal contact area of Group 2 did not differ significantly (*p* = 0.059); however, it tended to be lower in females than in males (Supporting Information S1: Figure 1 and Supporting Information S2: Figure 2).

**Table 3:** Comparison between men and women within each group.

Parameter	Group			
	G1	G2	G3	G4
Occlusal contact area	0.186	0.059	0.052	0.696
Occlusal force	0.512	0.001*	0.052	0.315
Tongue pressure	0.126	0.123	0.101	0.408
Lip pressure	0.082	0.017*	0.400	0.696
Masticatory efficiency	0.393	0.093	0.822	0.460

* *p* < 0.05.

### 3.8 Age‐Related Changes in Oral Function Parameters in Males and Females

In comparison with the analyses including all subjects (Figures 1a–e), analyses involving only males revealed no significant differences in certain oral function parameters (Supporting Information S1: Figure 1). The occlusal contact area and lip pressure exhibited no significant differences among the groups. Occlusal force and masticatory efficiency increased with an increase in age, exhibiting significant differences between Groups 1 and 4. Tongue pressure also increased with an increase in age, exhibiting significant differences between Groups 1 and 3 and Groups 1 and 4. Masticatory efficiency differed significantly between Groups 1 and 4.

Analyses involving only females revealed similar statistical differences as those shown in the analyses using all subjects to a certain extent (Supporting Information S2: Figure 2). The occlusal contact area in females differed significantly between Groups 2 and 4. The occlusal force also differed significantly between Groups 2 and 4. The tongue pressure in Group 1 differed significantly from that in Groups 3 and 4. Masticatory efficiency in Group 4 differed significantly from that in Groups 1 and 2.

### 3.9 Comparison of the Degree of Oral Function Development Among All Age Groups

Finally, we compared oral function development in each age group because we found incremental differences among the parameters. Figure 2 shows the degree of oral function development in each age group. Lip pressure was high even at an early stage of development and was stable during later stages. Masticatory efficiency, occlusal force, and tongue pressure constantly increased with age. Interestingly, the occlusal contact area temporally decreased during dental age IIIB. Statistical tests revealed that there were significant differences in oral function development in Groups 1 and 2 (Table 4). In Group 1, lip pressure and tongue pressure exhibited statistically significant differences. In Group 2, lip pressure and occlusal contact area showed statistically significant differences. These results suggest that lip pressure rapidly increases at an early stage of development until the age of 5 years and then remains stable during later stages. Other oral function parameters exhibited gradual increments during development; however, the occlusal contact area temporally decreased during dental age IIIB.

**Table 4:** Statistical comparison of the degree of oral function maturity at each age point.

Comparison of oral function	*p* value		
	G1	G2	G3
Lip pressure vs. occlusal contact area	1	0.021*	1
Lip pressure vs. masticatory efficiency	1	1	1
Lip pressure vs. occlusal force	0.456	0.845	1
Lip pressure vs. tongue pressure	0.002*	1	1
Occlusal contact area vs. masticatory efficiency	1	0.155	1
Occlusal contact area vs. occlusal force	1	1	1
Occlusal contact area vs. tongue pressure	0.11	0.239	1
Masticatory efficiency vs. occlusal force	1	1	1
Masticatory efficiency vs. tongue pressure	0.293	1	1
Occlusal force vs. tongue pressure	0.854	1	1

* *p* < 0.05.

## 4 Discussion

In this study, we discovered that oral function parameters exhibited different age‐related development patterns. The occlusal contact area, maximum bite force, tongue pressure, and masticatory efficiency gradually increased with age. Interestingly, the occlusal contact area tended to reduce temporarily between 5 and 12 years of age due to erupting lateral tooth replacement. Conversely, lip pressure was almost stable during this period. Although there were limited differences between males and females, similar parameter trends were observed for both sexes.

Our study had a limitation of the sample size. Although the power analysis indicated that our sample size was sufficient, some oral function parameters showed limited statistical significance. Perhaps, increasing the sample size may enhance the significance of age‐related changes; therefore, future studies with larger samples are desirable. For sex comparisons, the number of subjects was below the estimate, and a sample size increment is expected to yield more robust results.

The change in occlusal contact area from infancy to 20 years of age has been reported in several papers. According to a review by Koc, Dogan, and Bek, the occlusal contact area increases with age, changing significantly during the transition from primary to permanent dentition (Koc, Dogan, and Bek, 2010). Julien et al. compared the occlusal contact area between children aged 6–8 years and adults aged 18–25 years and found an increase in this parameter with age (Julien et al. 1996). Owais, Shaweesh, and Abu Alhaija investigated the maximum bite force and occlusal contact area of children in three dentition stages—primary, mixed, and permanent dentition—and found a gradual increase in these parameters throughout dental development (Owais, Shaweesh, and Abu Alhaija 2013). These research findings suggest that the occlusal contact area progressively increases from infancy to 20 years of age, with notable changes occurring during the transition from primary to permanent dentition (Julien et al. 1996; Koc, Dogan, and Bek 2010; Owais, Shaweesh, and Abu Alhaija 2013). This change is thought to reflect the development of occlusion associated with tooth eruption and craniofacial growth (Koc, Dogan, and Bek 2010). However, it is important to note that individual variations are substantial, and there is a wide range of occlusal contact areas even within the same age group (Julien et al. 1996; Koc, Dogan, and Bek 2010; Owais, Shaweesh, and Abu Alhaija 2013). In addition, the reported mean values may vary slightly depending on the measurement methods and study population (Koc, Dogan, and Bek 2010). Our data showed a reduction in occlusal contact area from Group 1 to Group 2, possibly attributed to the replacement of erupting lateral teeth in stage IIIB.

The maximum bite force tends to increase with age from infancy to adolescence (Kamegai et al. 2005). Kamegai et al. conducted a cross‐sectional study among 2594 Japanese children aged 3–17 years and found that this parameter increased significantly with age, suggesting a steady increase in bite force throughout childhood and adolescence (Kamegai et al. 2005). Owais, Shaweesh, and Abu Alhaija also investigated the maximum bite force in children aged 3–18 years (Owais, Shaweesh, and Abu Alhaija, 2012) and found that this parameter was significantly higher in boys than in girls across all age groups. In contrast, our data exhibited limited sex differences, with the maximum bite force only being significantly higher in males in Group 1. Braun et al. studied the maximum bite force in 1337 children and adolescents aged 6–18 years and found a significant increase in bite force with age (Braun et al. 1996). They also found that males had significantly higher bite forces than females, particularly after the age of 13 years. Overall, the increase in maximum bite force during this period is thought to be related to the growth and development of the masticatory system, including the jaw muscles and dental occlusion. As children grow, their masticatory muscles become stronger and their dental occlusion develops, leading to an increase in bite force.

Regarding the age‐related change in lip pressure, Koc, Dogan, and Bek measured this parameter in infants aged 0–5 years and reported that it increased with age; the mean lip pressure was 19.6 kPa for 0‐year‐olds and 36.1 kPa for 5‐year‐olds (Koc, Dogan, and Bek 2010). Hägg measured lip pressure in children and adolescents aged 7–19 years and reported that it increased with age (Hägg, Olgarsson, and Anniko 2008). Thüer measured lip pressure in children and adolescents aged 6–18 years and reported that it increased with age (Thüer 1999). As our data exhibited no age‐related increase in people aged 5–20 years, we hypothesized that lip pressure rapidly increases at an early stage of development until the age of 5 years, and it differs among individuals. Interestingly, our data also exhibited sex differences, with lip pressure values being higher in males in Group 2, possibly attributed to the difference in systemic muscle strength. Indeed, Neu et al. showed that muscle strength increased with age, with sex differences becoming more pronounced after the age of 11 years (Neu et al. 2002).

Tongue pressure tends to increase with age from infancy to adolescence (Van Dyck et al. 2015; Youmans, Youmans, and Stierwalt, 2009). Youmans, Youmans, and Stierwalt measured the maximum tongue pressure in healthy children aged 3–16 years and reported a rapid increase in tongue pressure during adolescence (Youmans, Youmans, and Stierwalt 2009). Van Dyck et al. also demonstrated an increase in tongue pressure with age in healthy children aged 6–11 years (Van Dyck et al. 2015). The increase in tongue pressure during this period is thought to be related to the growth and development of orofacial structures and the maturation of the neuromuscular system (Van Dyck et al. 2015; Youmans, Youmans, and Stierwalt 2009). As children grow, their oral and facial muscles become stronger, and their neural control of these muscles improves, leading to an increase in tongue pressure. Previous studies have suggested a positive correlation between oral cavity volume and tongue pressure, indicating that tongue pressure and function may influence palatal formation (Kinoshita, Kambara, and Kawamoto, 2007). Our data showed values similar to those reported in a previous study that classified subjects using Hellman's dental stages (Hashiguchi et al. 2017). Furthermore, a prior investigation reported that direct comparisons across age groups are possible when using an appropriately sized probe (Youmans, Youmans, and Stierwalt 2009). This suggests that standardized measurements can be obtained even in children with smaller oral cavities. Regarding sex differences, the study by Van Dyck et al. (2015) did not reveal any significant differences between boys and girls. Coincidentally, our data also indicated no significant difference in tongue pressure between males and females in all age groups. However, more research is needed to clarify the potential influence of sex on tongue pressure development in children and adolescents.

Regarding masticatory efficiency, Julien et al. investigated this parameter in children aged 6–18 years with a color‐changeable chewing gum and found that it improved significantly with age (Julien et al. 1996). Maki et al. conducted a study on the masticatory performance of children aged 7–12 years using a gummy jelly test and found a significant increase in it with age (Maki et al. 2001). Toro et al. investigated masticatory performance in children aged 6–17 years using a two‐color chewing gum mixing ability test and found that it increased significantly with age (Toro et al. 2006). They also found that masticatory efficiency was positively correlated with the number of occlusal contacts and negatively correlated with the number of missing teeth. Overall, the increase in masticatory efficiency during this period is thought to be related to the development of the masticatory system, including the eruption of permanent teeth, increase in bite force, and maturation of neuromuscular control. As children grow, their masticatory system becomes more efficient, enabling them to break down food particles more effectively. Our data were consistent with previous reports that exhibited a gradual increase in masticatory efficiency.

Our data revealed gradual augmentation of oral functions, except for lip pressure. These changes were consistent with those of other studies (Barrera et al. 2011; Braun et al. 1996; Fujita et al. 2018; Guo et al. 2021; Ichikawa et al. 2016; Julien et al. 1996; Maki et al. 2001; Owais, Shaweesh, and Abu Alhaija 2013). The occlusal contact area, occlusal force, tongue pressure, and masticatory efficiency increased with age; however, the occlusal contact area tended to decrease at the age of 9–12 years due to lateral tooth replacement in dental age IIIB. Coincidentally, the occlusal force exhibited stability at 5–12 years, possibly due to the high correlation between the occlusal force and occlusal contact area (Sasaki et al. 2023). Consistent with our results, Sonnesen reported that not only the number of teeth in contact but also the stage of dental eruption was significantly correlated with bite force (Sonnesen 2001). However, no longitudinal studies comparing the changes in occlusal contact area from the primary dentition to the permanent dentition exist. Therefore, further detailed explorations are necessary to clarify the change in occlusal contact area during permanent tooth succession. In contrast, lip pressure was stable during the examined age, and it would rapidly increase at an early stage of development until the age of 5 years. This finding was consistent with Koc's report (Koc, Dogan, and Bek 2010). This early development of lip pressure is presumed to be of high clinical significance in terms of preventing malocclusion because poor lip closure and low lip pressure are reported to be associated with malocclusion, including anterior open bite (Lambrechts et al. 2010). Furthermore, Grippaudo et al. suggested that appropriate lip pressure reduces the risk of developing anterior open bite due to oral habits such as finger sucking (Grippaudo et al. 2016). Another explanation for the stable lip pressure during our examined age would be the variance among individuals because Kinoshita, Kambara, and Kawamoto also reported limited differences among school grades due to the large variance (Kinoshita, Kambara, and Kawamoto 2007). Further exploration regarding why lip pressure is stable from 5 to 20 years is necessary. Therefore, further studies with larger samples should be conducted.

In conclusion, we discovered that oral function parameters exhibited different age‐related development patterns. Occlusal contact area, maximum bite force, tongue pressure, and masticatory efficiency gradually increased with age. Notably, the occlusal contact area tended to reduce temporarily from 5 to 12 years of age, which might be due to the replacement of erupting lateral teeth. In contrast, lip pressure was almost stable during the period measured in this study.

## Author Contributions

**Kiichiro Mizokami:** data curation, formal analysis, investigation, software, validation, visualization, drafted the manuscript. **Syunnosuke Tohyama:** formal analysis, project administration, software, supervision, validation, visualization. **Hiroyuki Kanzaki:** methodology, conceptualization, formal analysis, methodology, project administration, supervision, validation, writing–review and editing. **Yoji Sasaki:** data curation, investigation, methodology, validation. **Mao katayama:** data curation, investigation. **Minami Seki:** data curation, investigation. **Haruna Rikitake:** data curation, investigation. **Syoutaro Ueda:** data curation, investigation. **Toshiko Sekiya:** formal analysis, software. **Hiroshi Tomonari:** conceptualization, funding acquisition, methodology, project administration, resources, supervision, writing–review and editing.

## Conflicts of Interest

The authors declare no conflicts of interest.

## Supporting information

Supporting information.

Supporting information.

Supporting information.

## Data Availability

The data that support the findings of this study are available from the corresponding author upon reasonable request.
