# Supplementary figures and images for: Quantitative Comparison of Age‐Related Development of Oral Functions During Growing Age
Source: Clin Exp Dent Res. 2024 Nov 12;10(6):e70033. doi: 10.1002/cre2.70033 (PMC11556408; doi:10.1002/cre2.70033)

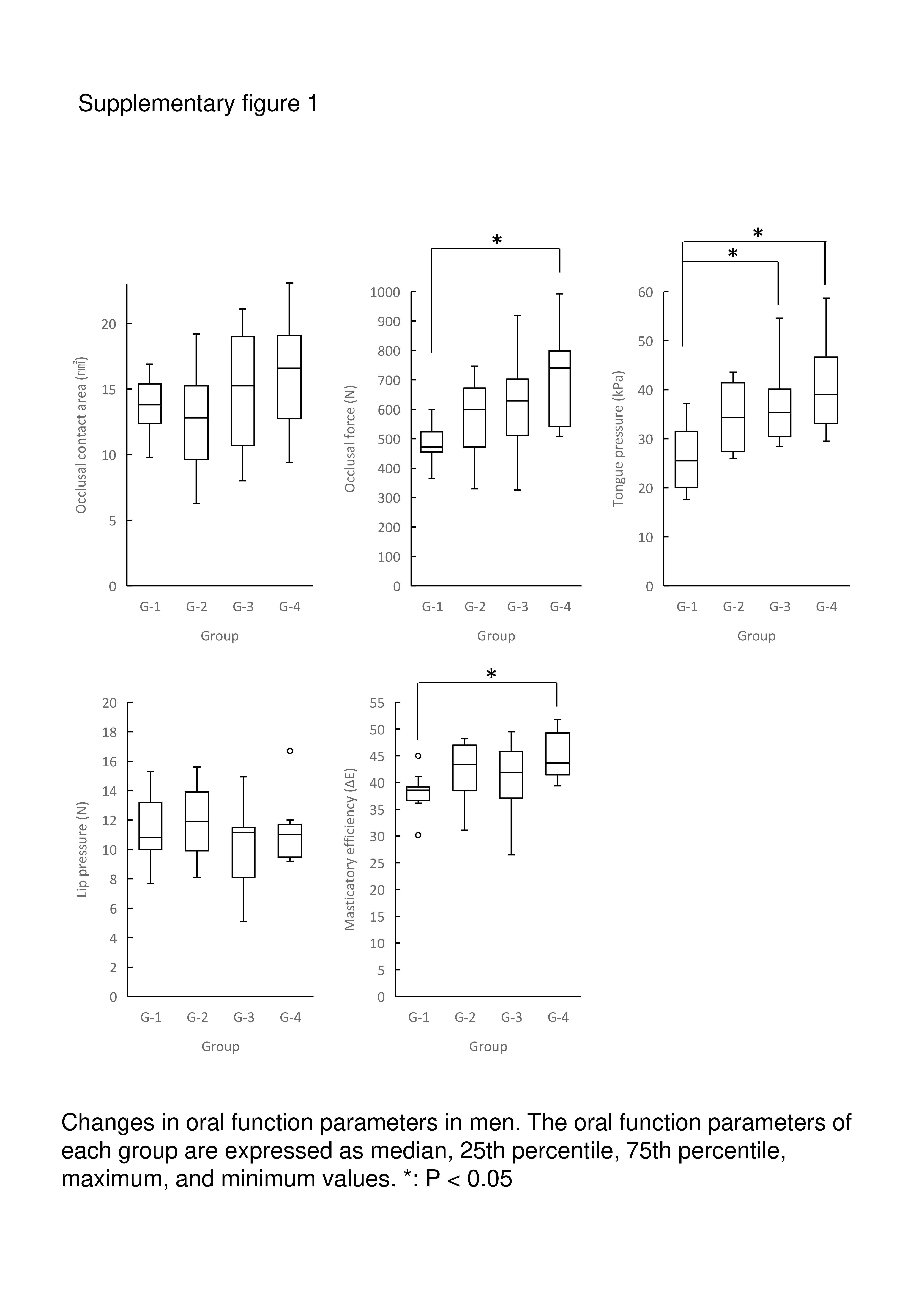

Supplement: Supplementary file 1 — Supporting information. [file CRE2-10-e70033-s001.tif]

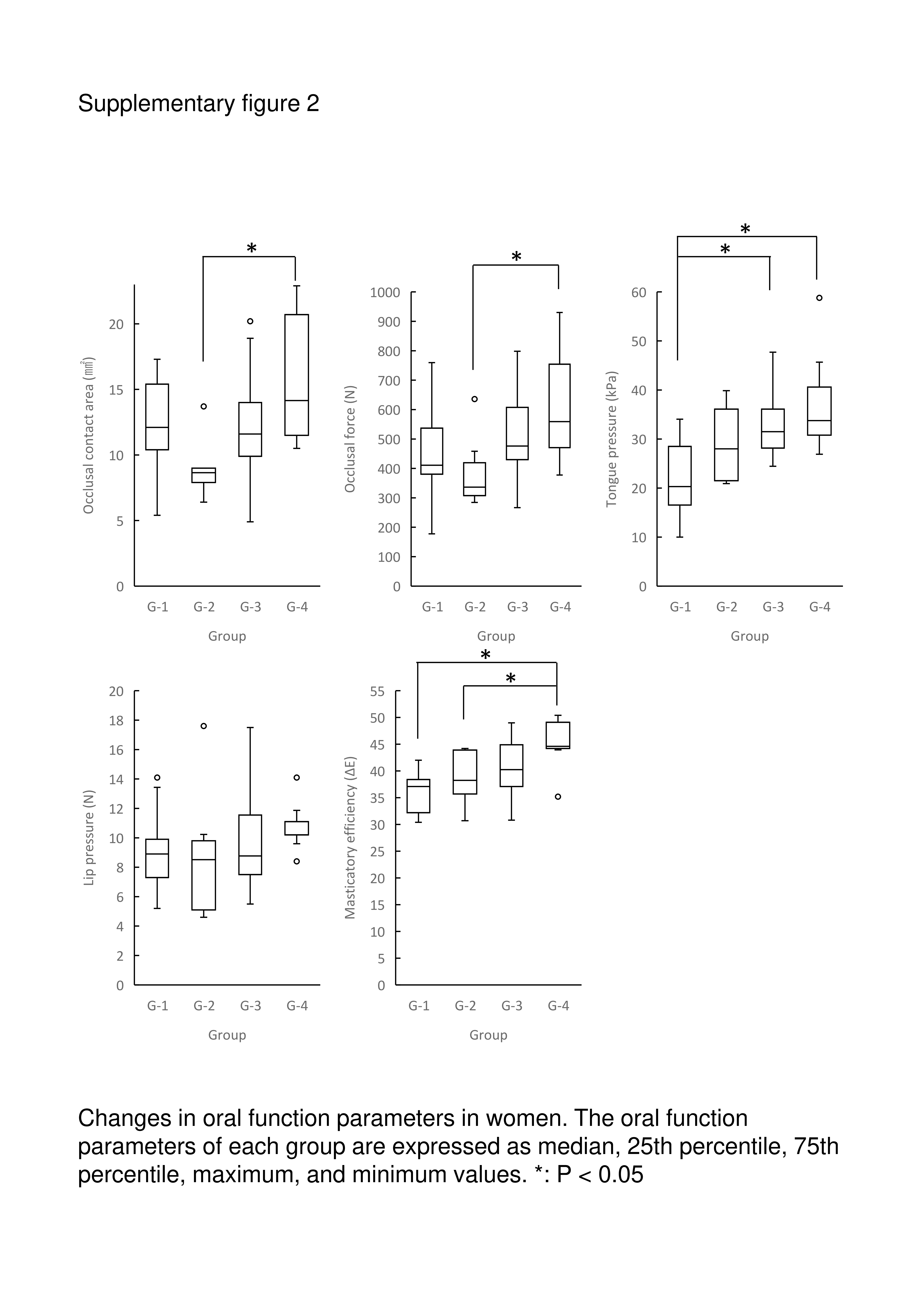

Supplement: Supplementary file 2 — Supporting information. [file CRE2-10-e70033-s002.tif]
