# Supplementary material for: Quantitative Comparison of Age‐Related Development of Oral Functions During Growing Age
Source: Clin Exp Dent Res. 2024 Nov 12;10(6):e70033. doi: 10.1002/cre2.70033 (PMC11556408; doi:10.1002/cre2.70033)
Supplement: Supplementary file 3 — Supporting information. [file CRE2-10-e70033-s003.docx]

Supplementary file

Figure legend for graphical abstract

This graphical abstract illustrates the age-related development of five major oral functions in subjects aged 5-20 years. Our findings reveal distinct developmental patterns across oral functions. Occlusal contact area, maximum bite force, tongue pressure, and masticatory efficiency demonstrated a gradual increase with age, while lip pressure remained relatively stable throughout the studied age range. Interestingly, we observed a temporary decrease in occlusal contact area between 5-12 years, likely attributable to the eruption of lateral teeth. Although some gender differences were noted, overall trends in oral function parameters were similar between males and females.

The figure on the right depicts the ranking of oral function development. Lip pressure showed the earliest maturation (ranked 1st), followed by bite force (ranked 2nd), and tongue pressure (ranked 3rd). This ranking highlights the differential rates of development among various oral functions during the growing years.

These results provide valuable insights into the maturation of oral functions during the growing years, highlighting the complex and varied developmental trajectories of different oral parameters. This understanding can inform clinical practices and future research in pediatric dentistry and oral health.

Figure legend for supplementary figure 1

Changes in oral function parameters in men. The oral function parameters of each group are expressed as median, 25th percentile, 75th percentile, maximum, and minimum values. *: P < 0.05

Figure legend for supplementary figure 2

Changes in oral function parameters in women. The oral function parameters of each group are expressed as median, 25th percentile, 75th percentile, maximum, and minimum values. *: P < 0.05
